# Effects of metalloprotease ADAMTS12 on cervical cancer cell phenotype and its potential mechanism

**DOI:** 10.1007/s12672-023-00776-2

**Published:** 2023-08-29

**Authors:** Ruanmin Zou, Ruihong Gu, Xinyu Tu, Jiani Chen, Songjun Liu, Xiangyang Xue, Wensu Li, Yuyang Zhang

**Affiliations:** 1 Department of Obstetrics and Gynecology, The First Affiliated Hospital, Wenzhou Medical University, Wenzhou, People’s Republic of China; 2 Department of Microbiology and Immunology, Institute of Molecular Virology and Immunology, Institute of Tropical Medicine, College of Basic Medicine, Wenzhou Medical University, Wenzhou, People’s Republic of China; 3 Department of Gynecology, Tongde Hospital of Zhejiang Province, Hangzhou, People’s Republic of China

**Keywords:** Cervical cancer, ADAMTS12, Migration, Invasion, TGF-beta

## Abstract

ADAMTS12 is a gene widely expressed in human tissues. We studied the expression level of ADAMTS12 in cervical cancer tissue and its relationship with clinicopathological features. We also explored the function of ADAMTS12 in cervical cancer cells and its underlying mechanisms. We found the higher expression level of ADAMTS12 in cancer tissues, which was associated with the worse overall survival rate. The immunofluorescence assay showed that the cytoplasm of cervical cancer cells is the main expression site of ADAMTS12. Overexpression of ADAMTS12 in HeLa and CaSki cells prominently promoted the cell proliferation, migration and invasion. We found that 2032 genes were correlated with ADAMTS12, which was mainly related to extracellular matrix, TGF-β signaling pathway. The phosphorylation levels of mTOR and 4E-BP1 were upregulated in ADAMTS12-overexpressing cells. Co-Immunoprecipitation combined with protein mass spectrometry showed that TGF-β signaling pathway-related proteins interacting with ADAMTS12 were screened from HeLa cells with ADAMTS12 overexpression. Therefore, we concluded that ADAMTS12 may affect the mTOR signaling pathway through the interacting with TGF-β1, and then affect the biological function of cervical cancer cells.

**Supplementary Information:**

The online version contains supplementary material available at 10.1007/s12672-023-00776-2.

## Introduction

The ADAMTS family is a disintegrin and metalloproteinase with thrombospondin motif proteins, consisting of 19 complex metalloproteases that can be secreted by tumor cells and stromal cells [1–4], and are involved in many human diseases, including cancer [5]. ADAMTS12, a member of the ADAMTS family, located in 5q35 of the human gene with a length of about 8572 bp and a predicted protein molecular size of about 177 kDa. Its structure includes: signal peptide, prodomain, catalytic domain, disintegrin-like domain, TSP1 like repeat sequence, cysteine-rich domain, the two spacers and the C-terminal TSP1-like repeat sequence [5–8]. In 2001, ADAMTS12 was first reported and has since been widely studied. ADAMTS12 has been confirmed to be expressed in a variety of human tissues [6] and plays a significant role in the pathological process of various diseases, such as tumor [8], inflammation [9, 10], arthritis [11, 12], intervertebral disc degeneration [13], angiogenesis [14], schizophrenia [15], and gonadal development [16]. In terms of tumor, the potential biological properties of ADAMTS12 are various in different tumors. In breast cancer, ADAMTS12 can suppress tumorigenesis when interacts with fibulin-2, its chaperone protein, but can also promote tumor development if fibulin-2 is absent [17]. ADAMTS12 has the effect of inhibiting colorectal cancer. It’s silent in cancer cells, but is activated in cancer stroma which may have the anti-tumor mechanism [18]. In esophageal squamous cell carcinoma, ADAMTS12 is one of the downstream regulatory targets of lncRNA HCG22, which plays a role in suppressing cancer progression [19]. In ovarian cancer, ADAMTS12 was overexpressed in intestinal metastatic tissues, suggesting that the high expression of ADAMTS12 gene predicts the occurrence of intestinal metastasis and invasive cancer [20]. The expression of ADAMTS12 gene was also significantly upregulated in metastatic carcinoma of renal cell carcinoma [21].

Cervical cancer is a common malignant gynecological tumor, but it is unclear whether ADAMTS12 is related to cervical carcinogenesis. In this research, we explored the relationship between the expression level of ADAMTS12 in cervical cancer tissues and clinicopathological characteristics, and verified that ADAMTS12 is an independent risk factor influencing the prognosis of cervical cancer patients. The malignant phenotype of ADAMTS12 in cervical cancer cell lines was verified by functional experiments. Bioinformatics analysis was performed to explore the biological processes that ADAMTS12 may be involved in. The association between ADAMTS12 and immune indicators was analyzed, and it was found that ADAMTS12 may play an oncogenic role via the TGF-β signaling pathway. In addition, ADAMTS12 regulated the malignant function of cervical cancer, which may also be related to B cells and macrophages. This article provides a preliminary basis for studying the mechanism of ADAMTS12 in cervical cancer, and it also suggests a new direction for gene therapy of cervical cancer, and proposes a theoretical basis for ADAMTS12 gene as a therapeutic target for cervical cancer.

## Materials and methods

### Experimental materials

HeLa, CaSki, SiHa cell lines were purchased from National Collection of Authenticated Cell Cultures (Shanghai, China). ADAMTS12 antibody for immunohistochemistry was purchased from Abcam Company, and ADAMTS12 antibody for Western blotting was purchased from Proteintech Company. pCMV-HA-C-ADAMTS12 plasmid was purchased from Beijing Qingke Biotechnology Co., LTD. Antifade mounting medium was purchased from Shanghai Beyotime Biotechnology Co., LTD. EdU cell proliferation kit was purchased from Guangzhou Ribobio Biotechnology Co., LTD.

### Patient data

Pathological tissues and clinical data were acquired from totally 382 cervical cancer patients who underwent surgical treatment at the Second Affiliated Hospital and the First Affiliated Hospital of Wenzhou Medical University from 2015 to 2018. The patients were followed up every 3 months in the first year and every 6 months in the second year. All patients signed written informed consent prior to participating. Overall survival (OS) was defined as the time from diagnosis to death. All 360 patients were at the stage of CIS-IIA2 and chose to receive surgical treatment precedingly. A total of 22 patients were at the stage of IIB-IV, 9 of them underwent radiotherapy and chemotherapy (CRT) after biopsy, 1 received CT or RT after biopsy, and remaining 12 received surgery first. Patients were divided into four groups for statistical analysis, namely CIS + I group, IIA1 group, IIA2 group and IIB-IV group. This study was designed in strict accordance with the international ethical guidelines for human biomedical research and approved by the Institutional Review Committee of the First Affiliated Hospital of Wenzhou Medical University.

### Transfection of plasmid into cervical cancer cells

Cervical cancer cells (5 × 10^5^) were plated into 6-well culture plates for 24 h before transfection. After 24 h later when cell adherence was complete, pCMV-HA-C-ADAMTS12 and pCMV-HA-C plasmid were respectively performed transient transfection into HeLa and CaSki cells according to lipo2000 specification.

### Preparation of tissue microarrays

Pathological wax blocks were sectionized by professional pathologists. After stained with eosin (HE), the perforated area was circled according to the staining results. Tissues were removed from the area using 1-1.5 mm diameter fine needles, arranged into a certain array, and then the array was embedded and sectionized. After the tissue microarrays were completed, HE staining was used to confirm that the tissues were at its point, and pathological confirmation was made at the same time. After verification, paraffin-sealed sections could be stored in a refrigerator at 4 ℃ for long-term use.

### Immunohistochemical staining analysis of tissue microarrays

Tissue microarrays were scanned on panoramic MIDI automatic digital slide (3DHISTECH, Budapest, Hungary). Staining intensity was independently evaluated by two pathologists based on the following scoring criteria: 0 (negative), 1 (weakly positive), 2 (medium positive) or 3 (strong positive). Simultaneously, the proportion of weakly/medium/strong positive staining area to the overall area was estimated and recorded as follows :0 (< 5%), 1 (5–25%), 2 (26–50%), 3 (51–75%) and 4 (> 75%). The total score was obtained by multiplying the percentage score with the staining intensity score. The two pathologists verified the results independently.

### Real-time quantitative PCR

Cell RNA extraction was performed and synthesis of cDNA by reverse transcription. Real-time fluorescence quantitative PCR was performed. ADAMTS12 primer sequences: forward primer: 5’–TAC ATC ATG GAG AAG AGA TAT GGG A − 3’, backward primer: 5’- TAG TTG GAA AAA TCC AGT CAG TCC-3’.

### Western blotting test and immunoprecipitation

After 48 h transfection, 100 µL mixed lysate were added to each well (mixed lysate ratio: PMSF and RIPA at the ratio of 1:100), and cells were lysated on ice for 5 min. After lysis, centrifuged at 13,000 rpm at 4℃ for 15 min, and the supernatant was sucked into a 1.5 m LEP tube. Westering blotting and immunoprecipitation were performed.

### Cell migration assay and invasion assay

Cells were counted with the Bulbous counting plate to adjust centration to 1 × 10^5^/200 µl. 200 µl cell suspension was added into the chamber while 600 µl 10% FBS complete medium was added beneath. Cells were cultured in a 37 ℃, 5% CO_2_ incubator. Then, cells were fixed staining and photographic analysis of Transwell results. For invasion assay, Matrigel was diluted with PBS to 0.5 mg/ml, and added 200 µl to each chamber, and placed in 5% CO_2_, 37 ℃ cell culture incubator for 2 h.

### EdU cell proliferation assay

After 5 × 10^5^ transfected cells were cultured for 24 h, EdU solution (reagent A) was added in cells for 2 h. PBS was used to wash off EdU solution twice for 5 min each. Then, added 50 µl 4% paraformaldehyde to each well and incubated at room temperature for 30 min. Discarded the fixator and added 50 µl 2 mg/ml glycine to each well, incubated in shaker for 5 min and cleaned with PBS for 5 min. EdU cell proliferation was performed to measure proliferation of cervical cancer cells.

### CCk-8 cell proliferation assay

2 × 10^3^ cells per well were seeded on 96-well plates. After 24 h transfection, cells were added with 100 µl CCK8 reagent to each well at 24 h, 48 and 72 h, and then incubated in a 37 ℃, 5% CO_2_ incubator for 1–3 h. The absorbance (OD value) was detected with a multifunctional microplate reader at 450 nm.

### Cell colony formation assay

The transfected cervical cancer cells were seeded into 6-well plates (800 cells/well) and cultured for about 2 weeks. When the cloned groups in the plate were visible to naked eye, we discarded medium and washed with PBS two times. Then cells were fixed with 4% paraformaldehyde for 15 min, stained with 0.4% crystal violet for another 15 min, rinsed slowly in clear water, dried at room temperature, and photographed.

### GO/KEGG function enrichment and GSEA pathway enrichment

We retrieved from the TCGA database (https://portal.gdc.cancer.gov/) and downloaded 309 cases of cervical cancer transcriptome data (FPKM value) and corresponding clinical data. The R package ggscatterstats was used for correlation analysis, filtering criteria: P < 0.05, correlation coefficient R > 0.3. Gene ontology (GO) and Kyoto Encyclopedia of Genes and Genomes (KEGG) were enriched by R package Cluster Profiler. Functional annotation clusters of GO biological processes, cellular components and molecular functions, and KEGG pathways were provided. Gene set enrichment analysis (GSEA) was adopted to explore the important functions of 2032 genes. In the current study, Hallmarks gene set was analyzed using molecular marker database. 1,000 genome permutations were performed to obtain a normalized enrichment score for each analysis.

### GO/KEGG enrichment of immune-related genes

Human immune-related genes were acquired from Imm Port project. These gene sets are broadly used in immune-related studies. We obtained 1811 genes from 17 immune-related pathways. 175 cervical cancer immune-related genes required for subsequent analysis were obtained by intersection of 2032 related genes with cervical cancer transcriptome data. GO and KEGG enrichment were performed using R packet clusterProfiler. Functional annotation clusters of GO biological processes, cellular components and molecular functions, and KEGG pathways were also provided.

### Statistical methods

In this study, GraphPad Prism 6.0 statistical software was used for data analysis. P < 0.05 was considered to be statistically significant difference between the two groups (*P < 0.05, **P < 0.01, ***P < 0.001). Stata/SE 15.0 statistical analysis software was used for descriptive statistics, univariate cox regression analysis and multivariate Cox regression analysis. R 3.6.3 was used for statistical analysis of biological information. Utilized packages include: Dplyr, Tibble, Cowplot, GGStatsPlot, GGplot2, clusterProfiler, Future. apply, enrichplot, ggrepel, and VennDiagram.

## Results

### Analysis of ADAMTS12 expression and prognosis in patients with cervical carcinoma by database

**Fig. 1:**
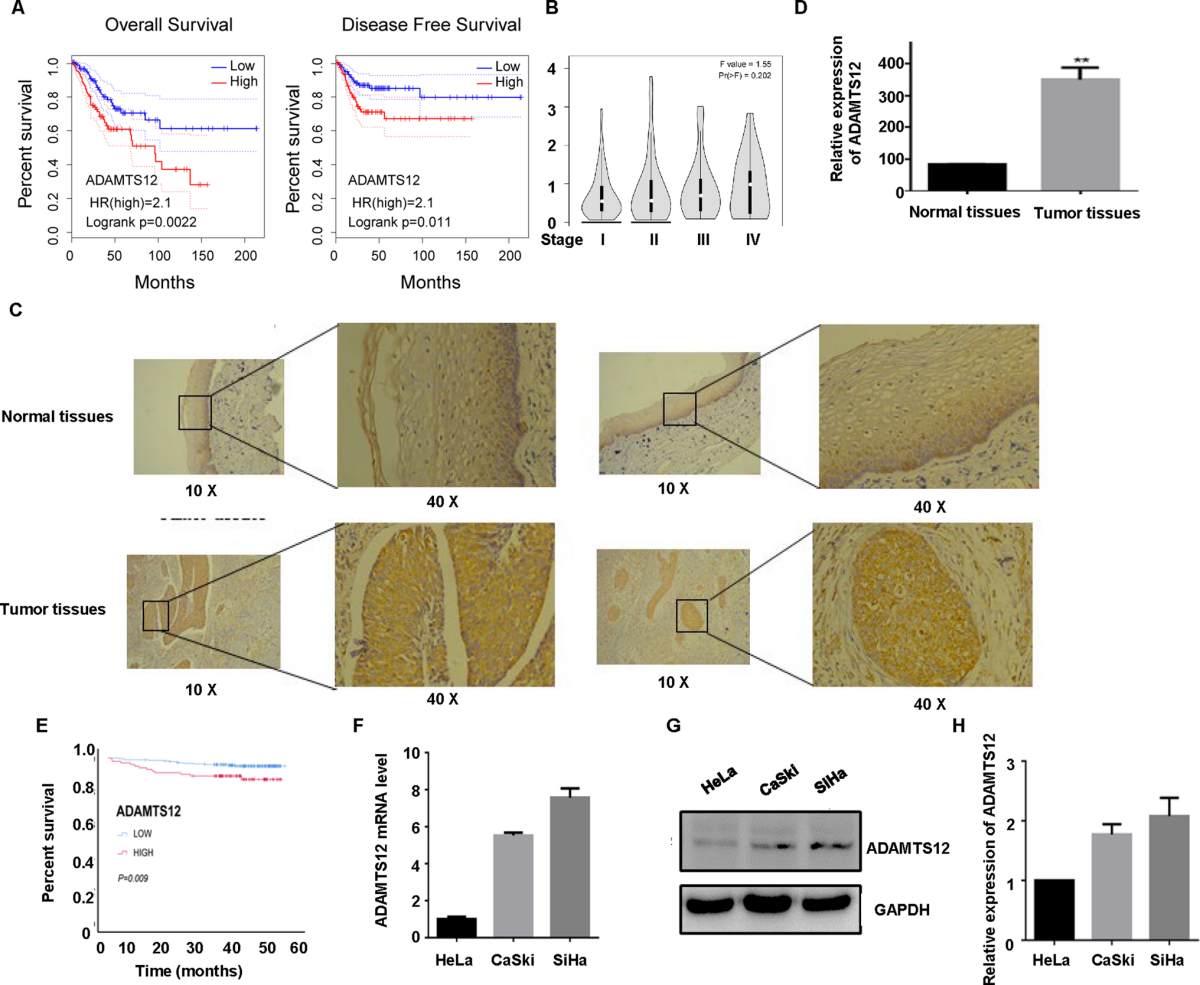
Expression of ADAMTS12 in cervical cancer. **A**: Cervical cancer patients were divided into two groups according to the expression level of ADAMTS12. Kaplan-Meier analysis was used to analyze the differences in overall survival rate (OS) and disease-free survival rate (DFS) between the two groups and draw survival curve. **B**: Expression level of ADAMTS12 in different stages of cervical cancer. **C–D**: Normal cervical tissues and cervical cancer tissues were stained by immunohistochemistry with ADAMTS12 specific antibody, and scanned. Blue staining indicated nucleus, and brown-yellow staining indicated positive staining. **E**: Kaplan-Meier survival curve was performed according to the scan results. Among the 382 cervical tissue microarrays, the higher the ADAMTS12 score, the worse the prognosis. **F**: ADAMTS12 expression at RNA level was detected by RT-PCR in cervical cancer cell lines. **G**: ADAMTS12 expression at protein level was detected by WB in cervical cell lines. **H**: Quantification of ADAMTS12 protein level in panel G

The relation between the expression level of ADAMTS12 and the overall survival rate as well as disease-free survival rate of patients in TCGA database was analyzed online on GEPIA website (http://gepia.cancer-pku.cn/). Median of ADAMTS12 expression level was used as the cut-off point to divided patients into high-expression group (n = 146) and low-expression group (n = 145). Kaplan-Meier analysis was used to plot the survival curve (Fig. 1A). Log-rank test suggested that the OS and DFS in low-expression group were significantly higher than that in high-expression group (OS: P = 0.0022, DFS: P = 0.011). Univariate Cox analysis was used to further test the difference in prognosis between the two groups, and results were consistent with K-M analysis in OS (HR = 2.1, P = 0.0027) and DFS (HR = 2.1, P = 0.015) (Fig. 1A). At the same time, we found that the ADAMTS12 level increased as tumor stage progressed (Fig. 1B). In conclusion, TCGA data analysis suggested that the higher the ADAMTS12 level, the worse the prognosis of cervical cancer patients.

### Correlation between ADAMTS12 protein level and clinicopathological features as well as prognosis in 382 cases of cervical cancer patients

Immunohistochemical staining of normal cervical tissues and cervical cancer tissues showed that ADAMTS12 was mainly expressed in the cytoplasm (Fig. 1C), and the expression of ADAMTS12 in cancer tissues was apparently higher than that in normal cervical tissues (quantified in Fig. 1D).We prepared cervical cancer tissue microarrays, which included 382 cases, detected the expression of ADAMTS12 in tumor tissues by immunohistochemistry, analyzed the correlation between ADAMTS12 expression and clinicopathological features. ADAMTS12 level was used as the dividing standard. We utilized ROC curve to choose the cut-off point to divide patients into two groups, high expression group and low expression group. We found significant differences in OS between the two groups of high and low expression (P = 0.009), and the higher expression level of ADAMTS12, the worse the prognosis (Fig. 1E), which was in line with the analysis results of TCGA database. Our data showed that a significant association was found between ADAMTS12 level and pathological type (P = 0.012), most patients with cervical squamous cell carcinoma (SCC) expressed low ADAMTS12, while in non-cervical squamous cell carcinoma that was high.

ADAMTS12 level was strongly correlated with tumor stage (P = 0.007). ADAMTS12 level was low in most patients with early cervical cancer (CIS + I), but high in most patients with stage II–IV, suggesting that the ADAMTS12 level increases with the progression of cervical cancer. Moreover, the level of ADAMTS12 was closely connected with the differentiation state of tumors (P = 0.004). Most of the patients with highly differentiated cervical carcinoma expressed low level of ADAMTS12, while most of the patients with poorly differentiated cervical carcinoma expressed a high level of ADAMTS12. There was also a noteworthy correlation between ADAMTS12 level and adjuvant chemoradiotherapy (P < 0.001). Compared with patients without adjuvant chemotherapy or radiotherapy, the proportion of patients with high ADAMTS12 level was higher in patients requiring adjuvant chemotherapy or radiotherapy. The proportion of patients that received adjuvant chemoradiotherapy with high levels of ADAMTS12 was even higher. However, ADAMTS12 expression level had no correlation with lymph node metastasis (Table 1).

**Table 1:** The clinical characteristics of patients according to the expression of ADAMTS12

Factor	Low	High	p-value
N	278	101	
Pathological type			0.012*
SCC	247 (88.8%)	79 (78.2%)	
Non-SCC	31 (11.2%)	22 (21.8%)	
Stage			0.007**
CIS + I	188 (67.6%)	49 (48.5%)	
IIA1	59 (21.2%)	36 (35.6%)	
IIA2	18 (6.5%)	9 (8.9%)	
IIB-IV	13 (4.7%)	7 (6.9%)	
Differentiation			0.004**
High	101 (36.3%)	19 (18.8%)	
Middle	88 (31.7%)	39 (38.6%)	
Low	89 (32.0%)	43 (42.6%)	
Lymph gland			0.32
Negative	241 (86.7%)	83 (82.2%)	
Positive	37 (13.3%)	18 (17.8%)	
Chemoradiotherapy			< 0.001***
Non-CRT	155 (55.8%)	35 (34.7%)	
CT or RT	52 (18.7%)	23 (22.8%)	
CRT	71 (25.5%)	43 (42.6%)	

Statistically significant***(p < 0.001),**(p < 0.01),*(p < 0.05)

### Univariate and multivariate Cox regression analysis of ADAMTS12 and clinicopathological features

In order to explore the relationship between ADAMTS12 protein level and patient survival, univariate and multivariate Cox regression analysis were adopted. In univariate analysis, ADAMTS12 expression level was a risk factor for OS (HR = 2.745, P = 0.012). In addition, high FIGO stage, low differentiation state, positive lymph node metastasis, and receiving adjuvant chemoradiotherapy were significantly associated with low survival. These important clinicopathological features were further analyzed in multivariate Cox regression analysis. Although pathologic type showed no significant correlation with prognosis (P = 0.730), it was included in multivariate Cox regression analysis considering its importance in clinical diagnosis and prognosis. Multivariate cox regression analysis indicated that ADAMTS12 level, high FIGO stage and positive lymph node metastasis were independent risk factors. We concluded that ADAMTS12 level was an independent risk factor for survival and prognosis of cervical cancer patients (HR = 2.339, P = 0.043) (Table 2).

**Table 2:** cox regression analysis of the risk score and clinical variables

Variables	Univariate-cox			Multiple-cox		
	P	Haz. Ratio	95% CI	P	Haz. Ratio	95% CI
ADAMTS12	0.012	2.745	1.251–6.022	0.043	2.339	1.026–5.333
Pathological type						
SCC		1.000			1.000	
Non-SCC	0.730	0.809	0.242–2.702	0.700	1.287	0.357–4.642
Stages						
CIS + I		1.000			1.000	
IIA1	0.003	5.228	1.787–15.300	0.163	2.350	0.707–7.806
IIA2	0.002	7.733	2.076–28.804	0.069	3.879	0.901–16.703
IIB-IV	0.001	17.698	5.366–58.369	0.001	11.191	2.820-44.407
Differentiation						
High		1.000			1.000	
Middle	0.716	1.394	0.233–8.342	0.508	0.528	0.079–3.510
Low	0.002	9.576	2.238–40.970	0.151	3.317	0.647–17.018
Lymphgland						
Negative		1.000			1.000	
Positive	0.001	9.804	4.402–21.833	0.002	4.524	1.769–11.572
Chemoradiotherapy						
Non-CRT		1.000			1.000	
CT or RT	0.040	3.757	1.060-13.313	0.602	1.440	0.366–5.662
CRT	0.001	6.746	2.238–20.336	0.677	0.748	0.192–2.925

### Expression of ADAMTS12 in cervical cancer cell lines

**Fig. 2:**
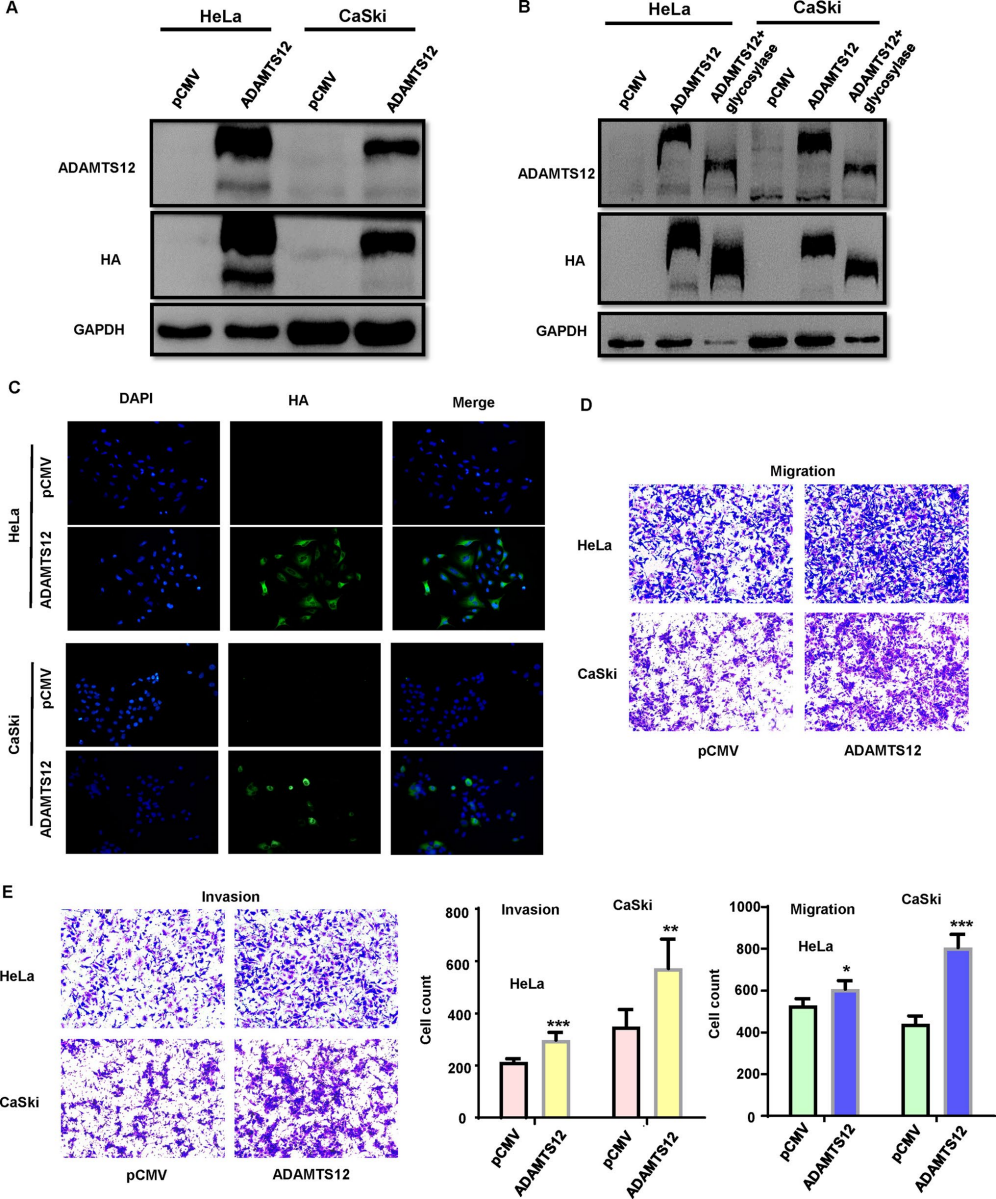
Overexpression of ADAMTS12 causes protein glycosylation, migration and invasion. **A**: After transfection of ADAMTS12 plasmid into cervical cancer cells HeLa and CaSki, ADAMTS12 protein expression was significantly increased. **B**: The changes of ADAMTS12 protein in the blank group, overexpression group, and glycosylase treatment group in HeLa and CaSki cells. **C**: ADAMTS12 was mainly located in cytoplasm of HeLa and CaSki cells. **D–E**: pCMV-ADAMTS12 plasmid was transfected into HeLa and CaSki cells, and Transwell experiments showed that upregulation of ADAMTS12 gene could promote cell migration and invasion (*P < 0.05, **P < 0.01, ***P < 0.001)

We designed the RT-qPCR primers for ADAMTS12 based on NCBI database data, the product was 159 bp. RT-qPCR was used to detect ADAMTS12 in cervical cancer cell lines (HeLa, CaSki, and SiHa) at RNA levels (Fig. 1F). The expression level of ADAMTS12 in cervical cancer cell lines was detected at the protein level (Fig. 1G, H). According to NCBI database data, we constructed an overexpressed recombinant plasmid pcMV-ADAMTS12-HA with HA label. The full-length gene was commissioned to be synthesized by Optimus Biologica, and the constructed plasmid was completely consistent with the desired target sequence by sequencing comparison. HeLa and CaSki cells were selected to verify the effectiveness of overexpression of the constructed recombinant plasmid in cells. pCMV-HA-C plasmid was used as the blank control group, and cell protein was extracted after 48 h transfection. HA labeled antibody and ADAMTS12 rabbit polyclonal antibody were used as the primary antibody for WB detection. In comparison with the control group, ADAMTS12 protein was significantly expressed in the experimental group (Fig. 2A).

ADAMTS12 protein was deeply expressed at 230 KDa band, and weakly expressed at 178 KDa bands. Considered that the protein might be glycosylated, we utilized glycosylated enzyme according to the band position of HA labeled antibody. Afterwards, WB showed that in experimental group 230 KDa decreased significantly close to the 178 KDa band (Fig. 2B). The results suggest that ADAMTS12 can be successfully overexpressed in vitro cervical cancer cells, and ADAMTS12 may be modified in vitro, resulting in protein molecular weight increase. In order to figure out subcellular localization of ADAMTS12 overexpression in vitro, cell immunofluorescence assay was used. Plasmids pCMV-HA and pCMV-adamts12-HA were transfected into cervical cancer cell lines (HeLa and CaSki), respectively. The results displayed that ADAMTS12 was mainly located in cytoplasm (Fig. 2C).

### Oncogenic function of ADAMTS12 at the cellular level

Next, two cervical cancer cell lines, HeLa and CaSki, were selected for cell functional experiments. The recombinant plasmid ADAMTS12 was overexpressed in HeLa and CaSki cells, and the results of transwell migration and invasion experiment indicated that compared with the control group, the migration and invasion ability of ADAMTS12 overexpressed cells increased enormously (P < 0.05) (Fig. 2D–E). Therefore, we believe that ADAMTS12 can promote the migration and invasion ability of cervical cancer cells.

**Fig. 3:**
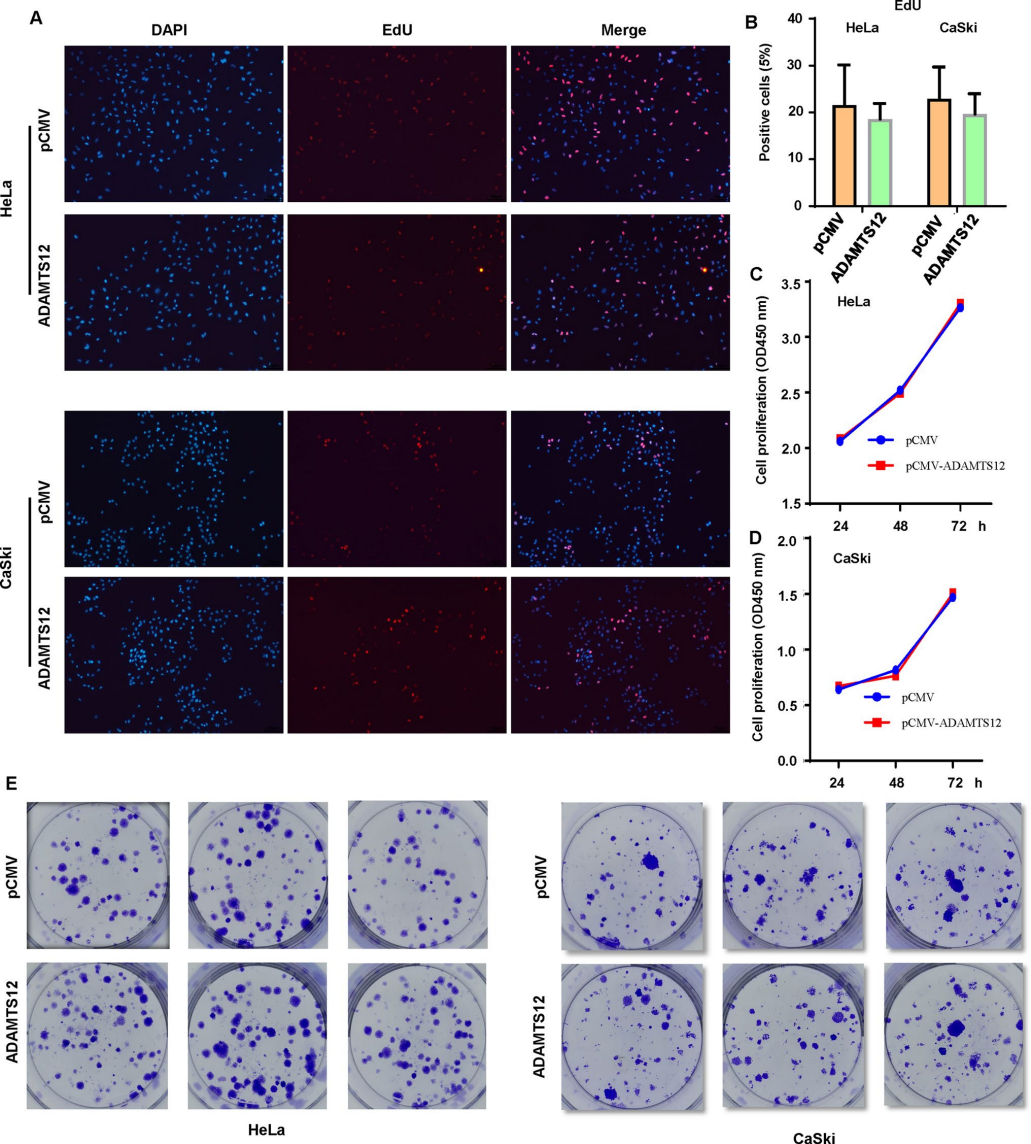
Proliferation of cervical cancer cells with pCMV-ADAMTS12 overexpressed plasmid infected. **A**: The plasmid pCMV-ADAMTS12 was transfected into HeLa and CaSki cells of cervical cancer. EdU proliferation experiment showed that the plasmid pCMV-ADAMTS12 could not promote cell proliferation ability. **B**: Quantification of EdU proliferation experiment results. **C**–**D**: CCK8 results showed that the plasmid pCMV-ADAMTS12 could not promote the proliferation ability of cells. **E**: The results of clonogenesis experiment showed that overexpression of plasmid pCMV-ADAMTS12 could not promote the clonogenesis ability of cells

In order to research the effects of ADAMTS12 on proliferation of cervical cancer cells, we performed EdU cell proliferation assay, clonogenesis and CCK8 to detect the proliferation indexes of HeLa and CaSki cell transfected with ADAMTS12 plasmid. The results showed no significant difference in EdU proliferation experiment of HeLa and CaSki cells (Fig. 3A, B). HeLa and CaSki cells were also used for CCK8 and clonogenesis experiment. The results showed that the overexpressed pCMV-ADA MTS12 group also had no significant difference for proliferation and colony formation from the blank control group (Fig. 3C–E).

### Explore the carcinogenic mechanism of ADAMTS12 through bioinformatics

**Fig. 4:**
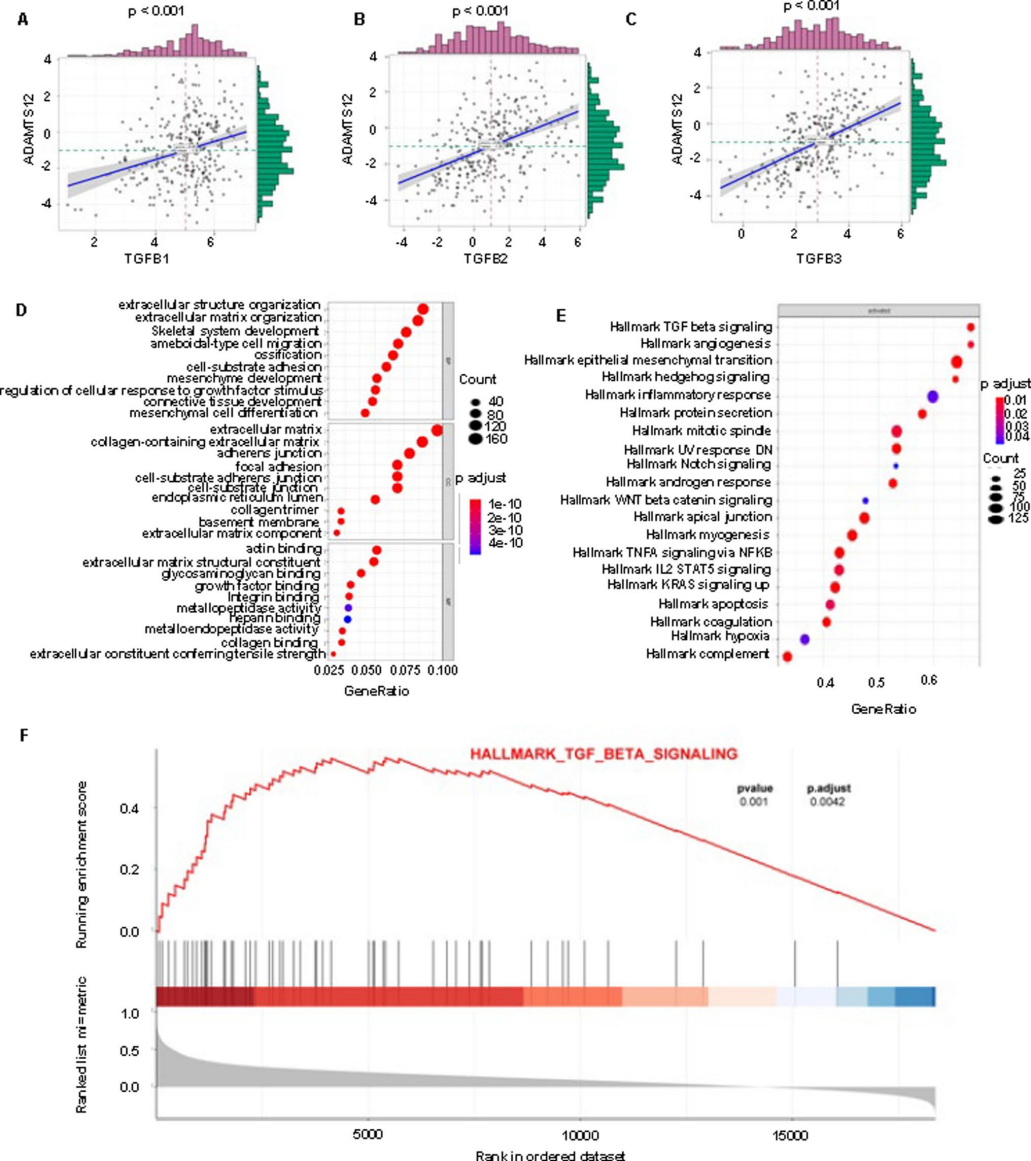
Enrichment analysis of ADAMTS12 related genes using TCGA database. **A**–**C**: Single gene correlation analysis of ADAMTS12 was performed using cervical cancer data in the TCGA database (R > 0.3, P < 0.05), and total of 2032 related genes were obtained. This graph shows the correlation between three random genes and ADAMTS12. **D**: GO analysis was performed using 2032 correlated genes screened from the TCGA database. **E**: GSEA analysis was performed to select 20 related pathways from 50 typical HALLMARK gene pathways. **F**: TGF-b signaling pathway was illustrated

To seek the regulatory pathway of ADAMTS12 in cervical cancer, we used the transcriptome data of 306 cases of cervical cancer in the TCGA database and conducted a single-gene correlation analysis of the ADAMTS12 gene by R software. As shown in Fig. 4A, C, the correlation coefficient r of ADAMTS12 with TGF-β1, TGF-β2, and TGF-β3 genes was 0.31, 0.46, and 0.52 (P < 0.001), respectively. A total of 2032 ADAMTS12-related genes were screened out (R > 0.3, P < 0.05). GO enrichment analysis showed that ADAMTS12-related genes were mainly enriched in the extracellular matrix, especially in the collagen-containing extracellular matrix (Fig. 4D). Their molecular function is mainly to bind actin and participate in formation of extracellular matrix. The most abundant biological processes are involved in tissue formation of extracellular structure and extracellular matrix, which are closely related to the function of promoting cervical cancer cell migration in vitro. We introduced the above-mentioned 2032 related genes into HALLMARK data set for GSEA analysis. After sequencing all pathways according to gene proportion, the pathways with P > 0.05 were removed, and the top 20 pathways in the sequencing table were selected, mainly including transforming growth factor (TGF-β) signaling pathway (Fig. 4F). These results indicated that ADAMTS12 may promote the migration and invasion of cervical cancer cells by interacting with some proteins in the TGF-β signaling pathway.

### Effects of ADAMTS12 on mTOR signaling pathway

**Fig. 5:**
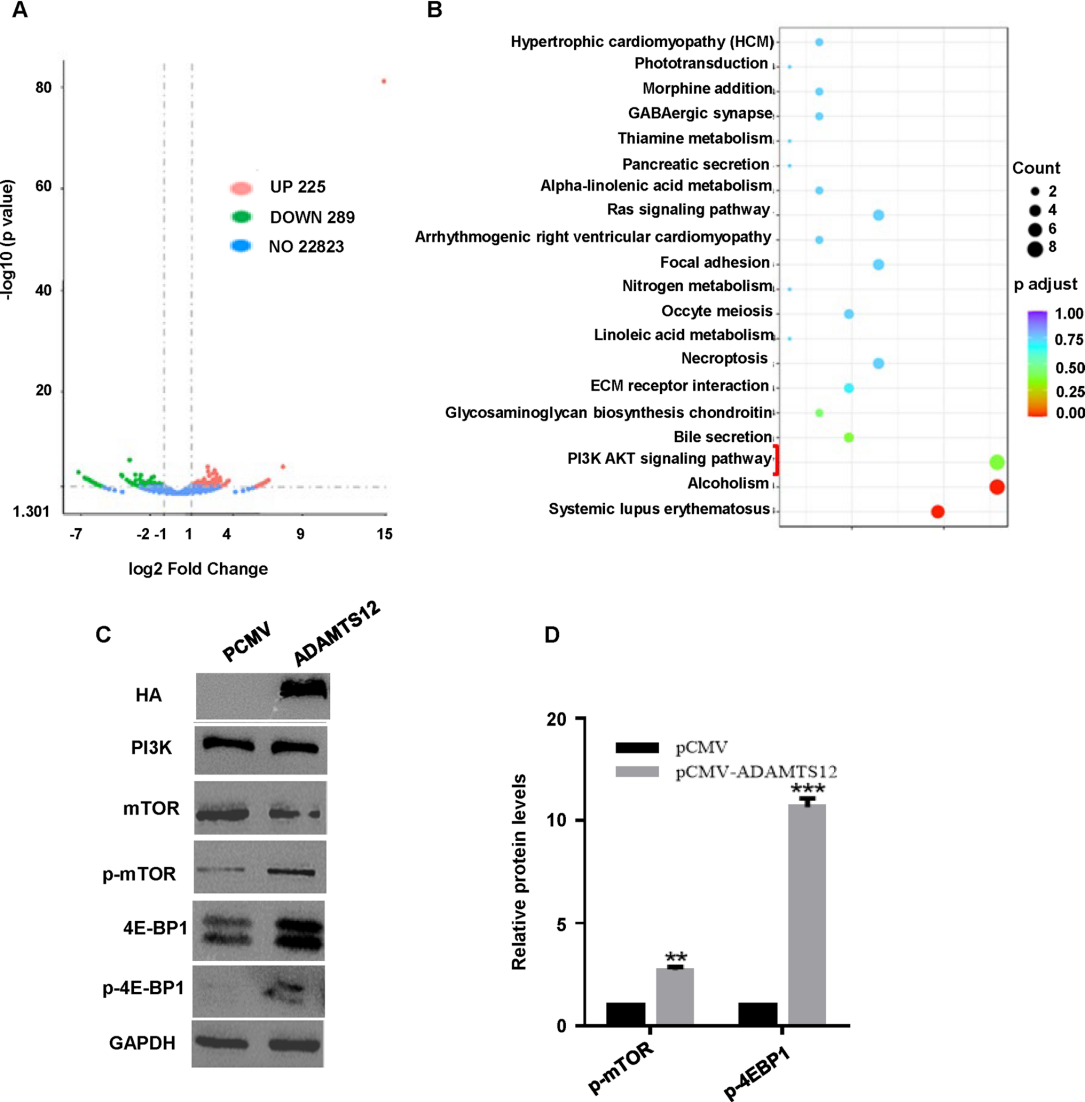
WB results verified the key protein molecules of mTOR signaling pathway. **A**: According to the results of transcriptome sequencing, the volcano map showed that the upregulation of ADAMTS12 gene in HeLa cells resulted in upregulation of 225 genes and downregulation of 285 genes. **B**: KEGG pathway enrichment analysis was performed using these upregulated and downregulated genes. **C**: Upregulation of ADAMTS12 in HeLa cells significantly upregulated phosphorylated mTOR and phosphorylated 4E-BP1 protein levels. **D**: Quantification of protein expression in panel C

To further corroborate the connection between ADAMTS12 and TGF-β signaling pathway, we overexpressed ADAMTS12 in HeLa cells for transcriptome sequencing analysis. As shown in volcano diagram, we found that after the upregulation of the ADAMTS12 gene in HeLa cells, 225 genes were upregulated and 285 genes were downregulated (Fig. 5A). Next, clusterProfiler was used for KEGG pathway enrichment analysis of these genes, which was enriched to 20 signaling pathways, among which the downstream pathway of TGF-β-- PI3K/mTOR signaling pathway was the most significantly affected (Fig. 5B).

PI3K/mTOR signaling pathway is a bridge between extracellular signal and intracellular response and takes an important part in the development and progression of tumors [22, 23]. The molecular expression level and phosphorylation levels of PI3K, mTOR, and 4EBP1D proteins in cervical cancer cells with the ADAMTS12 overexpression group were detected by WB. The results showed that upregulation of ADAMTS12 expression significantly upregulated the levels of phosphorylated mTOR and phosphorylated 4E-BP1 in HeLa cells (Fig. 5C, D). It is speculated that ADAMTS12 may regulate the invasion and migration of cervical cancer cells by affecting the mTOR signaling pathway.

### The mechanism of ADAMTS12 promoting cancer preliminarily explored by co-immunoprecipitation combined with protein mass spectrometry

**Fig. 6:**
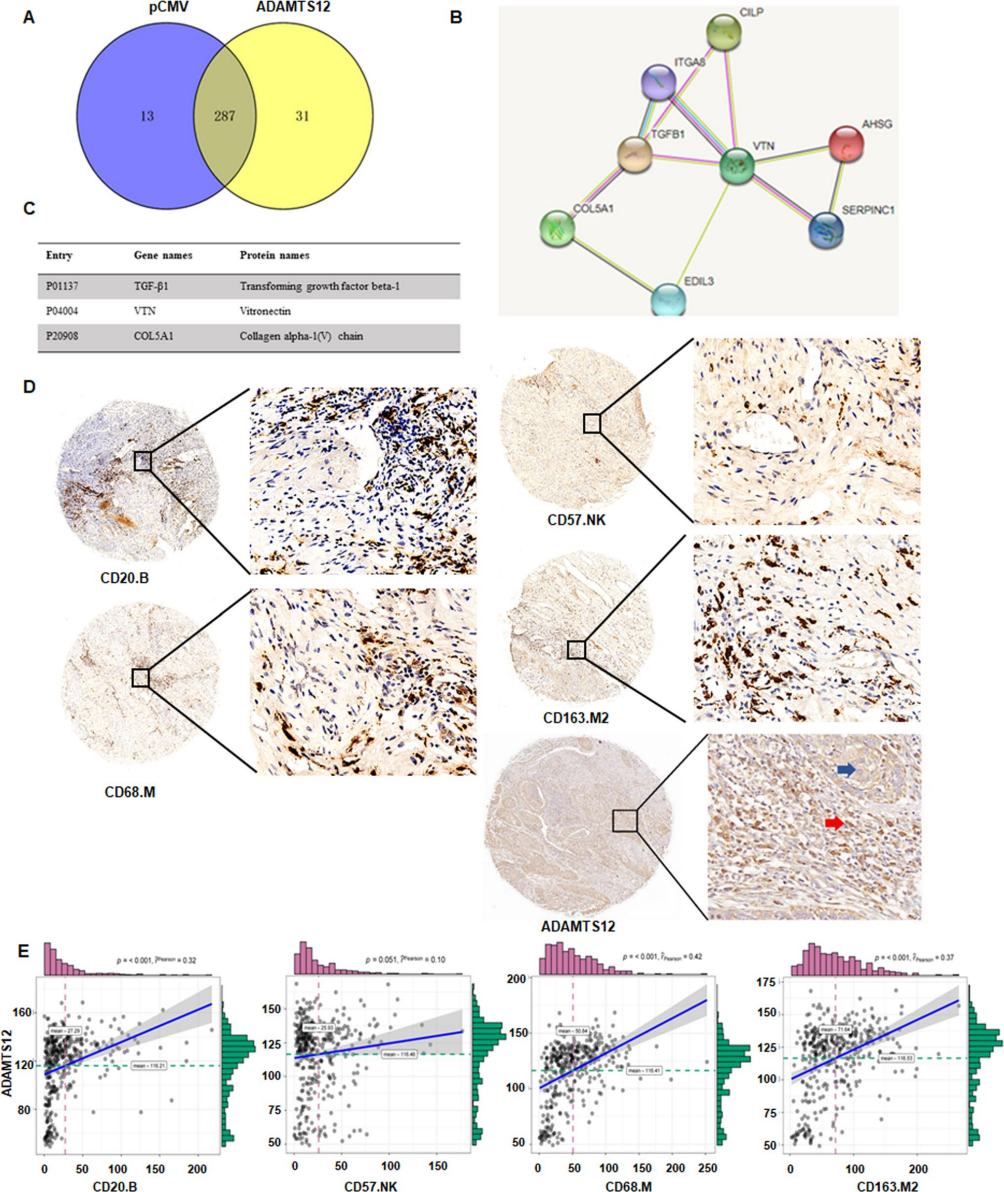
Upregulated ADAMTS12 gene in HeLa cells for co-immunoprecipitation combined with mass spectrometry. **A**: Venn diagram shows the amount of protein detected in both groups. **B**: Protein-protein interaction analysis was performed on some differentially expressed genes using the STRING database. **C**: The result of B was illustrated. **D**: Cervical cancer microarray was stained with specific antibodies of CD20, CD57, CD68, CD163 and ADAMTS12 by immunohistochemistry method, and scanned by the scanner. Blue staining indicated nucleus and brown-yellow staining indicated positive staining. **E**: The expression levels of ADAMTS12 were positively correlated with CD20 + B, CD68 + M, and CD163 + M2 cells in cervical cancer tissue microarray, but not with CD57 + NK cells

To further explore the mechanism of ADAMTS12 promoting the migration and invasion of cervical cancer cells, the ADAMTS12 recombinant plasmid was transfected into HeLa cell lines, and proteins were extracted for co-immunoprecipitation combined with mass spectrometry analysis. By drawing a superimposed Venn diagram of regulated proteins in the two groups of cells (Fig. 6A), 31 proteins that may interact with ADAMTS12, such as TGF-β1, VTN, and COL5A1, were screened. STRING analysis further revealed that the ADAMTS12 interacting protein had TGF-β1-VTN centered interaction (Fig. 6B–C). At present, studies have proved that the TGF-β signaling pathway plays a role of tumor suppressor in the early stage of tumor development; however, in the middle and late stages, this signaling pathway can play a role in cancer promotion [24–26]. These results further suggest that ADAMTS12 may affect the biological function of cervical cancer through TGF-β signaling pathway.

### Analyze the association between ADAMTS12 and immunity

Co-Immunoprecipitation combined with mass spectrometry showed that ADAMTS12 may affect the biological function of cervical cancer through TGF-β signaling pathway, which is a considerable mechanism affecting tumor immune microenvironment. In addition, GO analysis of ADAMTS12-related genes in TCGA data was closely related to cell-matrix, so we further explored the correlation between ADMATS12 and infiltration of immune cells in the matrix. H-score was used to analyze the expression of CD20, CD57, CD68 and CD163 in cervical cancer microarray (Fig. 6D). These four CD molecules represent CD20 + B cells, CD57 + NK cells, CD68 + M cells, and CD163 + M2 cells. The correlation between 4 immune cells and ADAMTS12 was analyzed by Pearson (Fig. 6E). ADAMTS12 was positively correlated with CD20 + B cells (R = 0.32, P < 0.001). There was no significant correlation between ADAMTS12 and CD57 + NK cells (R = 0.10, P < 0.051). ADAMTS12 was positively correlated with CD68 + M cells (R = 0.42, P < 0.001). ADAMTS12 was positively correlated with CD163 + M2 cells (R = 0.37, P < 0.001). These results suggest that ADAMTS12 may also participate in the development of cervical cancer through B cells, macrophages, and other immune cell infiltration.

## Discussion

ADAMTS12 has been extensively studied since it was first reported. Previous studies revealed that ADAMTS12 has a great biological role in breast cancer, colorectal cancer, esophageal cancer, and other different tumors. We have reason to speculate that ADAMTS12 may play a significant role in cervical cancer, so it is necessary to study the role of this gene in cervical cancer. For this study, the clinical data of cervical cancer patients in TCGA database were analyzed. ADAMTS12 level increased gradually with the progression of the disease, and higher expression level was interrelated with shorter survival and recurrence survival. We detected the expression level of ADAMTS12 gene in cervical cancer tissues and normal cervical tissues by immunohistochemistry, and found that the expression level of ADAMTS12 gene in cervical cancer tissues was evidently higher than that in normal cervical tissues, and the analysis results of 382 cervical cancer microarray also suggested that the high expression level of ADAMTS12 predicted a poorer prognosis.

Therefore, we further explored the relationship between ADAMTS12 and cervical cancer phenotype. Previous studies have found that some factors may affect the prognosis of cervical cancer, such as FIGO stage [27], degree of differentiation [28], lymph node metastasis [28, 29], radiotherapy and chemotherapy [30], pathological types [31], are considered prognostic factors for cervical cancer and are widely used in research. We found a relationship between ADAMTS12 expression levels and the clinicopathological features of cervical cancer, which confirmed our previous inference, and we conducted further studies on its predictive ability. First, we analyzed ADAMTS12 levels in 382 cervical cancer patients and found that ADAMTS12 levels were higher in tumors with a higher degree of malignancy, including patients with high FIGO stage, poorly differentiated status, and pathological type of non-squamous cell carcinoma. The higher FIGO stage was the result of tumor progression, which is consistent with the conclusion that ADAMTS12 levels increase with tumor progression, suggesting that ADAMTS12 has the potential to predict tumor progression. In addition, ADAMST12 levels also increased in cervical cancer tissues of patients receiving adjuvant concurrent chemoradiotherapy. Although whether a patient received adjuvant concurrent chemoradiotherapy was not a result of tumor progression, it indirectly implied tumor malignancy, as patients with higher malignancy were more likely to receive combined chemoradiation. Although these analyses suggest that ADAMTS12 levels may predict tumor malignancy, it cannot be determined whether ADAMTS12 is directly related to tumor malignancy or indirectly related to tumors due to factors such as stage, differentiation, and adjuvant chemoradiotherapy. Therefore, multiple regression analysis was needed to determine whether ADAMTS12 level was an independent risk factor.

Clinical indicators such as FIGO stage, tumor differentiation type, and lymph node metastasis have been used by scholars to establish clinical prediction models, and the constructed clinical prediction models can predict the 5-year survival rate of cervical cancer patients [32–34]. In this study, we examined whether the expression level of ADAMTS12 could be a new independent risk predictor. We used Cox hazard proportional regression model for analysis, ADAMTS12 level, FIGO stage, tumor differentiation type, lymph node metastasis, adjuvant chemoradiotherapy treatment, and pathological type were all used as potential covariates to participate in univariate Cox regression analysis. The results displayed that all these factors, except pathological type, had a significant association with survival and were used for multiple regression analysis. But considering the importance of pathological type in clinical diagnosis and prognosis, pathological type was also included in multiple regression analysis. Multivariate cox regression analysis showed that ADAMST12 level was an independent risk factor for cervical cancer (HR = 2.339 (1.026–5.333, P = 0.043), which could be used as a clinical predictor.

According to the ADAMTS12 gene sequence in NCBI database, pCMV was selected as the vector to construct the overexpression plasmid. To verify the cancer-promoting phenotype of ADAMTS12 in experiments, we selected HeLa and CaSki to conduct cell functional experiments. After transfection with ADAMTS12 plasmid, the migration and invasion of cervical cancer cells were significantly enhanced, but the cloning and proliferation ability were not affected. These results are consistent with expectations, confirming that exogenous overexpression of ADAMTS12 can promote the malignant phenotype of cervical cancer cells.

Next, we screened 353 interacting proteins, including TGF-β1 protein, by up-regulating ADAMTS12 gene in HeLa cells and performing co-immunoprecipitation mass spectrometry analysis. At present, studies have shown that in the early stage of tumorigenesis, TGF-β induces cell cycle arrest and apoptosis to inhibit the occurrence and growth of primary tumors. In the late stage of tumor progression, when the tumor cytokine TGF-β signaling pathway is inactivated or the cell cycle is abnormally regulated and becomes resistant to the growth inhibition of TGF-β, the effect of TGF-β has a pro-tumor effect.

To explore the cancer-promoting mechanism of ADAMTS12, we first began to search through the bioinformatics level. We downloaded sequencing data from the TCGA database. To search for genes closely related to ADAMTS12, 2032 genes were obtained by using the spearman method and statistical processing with R version 3.6.3. GO analysis of these genes showed that the main enriched biological processes were extracellular structure organization and extracellular matrix, which were closely related to their function of promoting the migration and invasion of cervical cancer cells in vitro. In addition, through GSEA enrichment analysis, the TGF-β signaling pathway was enriched. Therefore, we speculate that ADAMTS12 may affect TGF-β1 expression to participate in the progression of cervical cancer [24–26, 35].

According to the transcriptome sequencing after ADAMTS12 gene upregulated in HeLa cells, results suggested that 225 genes were upregulated and 289 genes were downregulated. These differential genes were enriched to PI3K/mTOR signaling pathway by KEGG analysis. Since PI3K is downstream of TGF-β signaling pathway, we speculated that ADMTS12 might affect the downstream signaling pathway through interacting with TGF-β. PI3K signaling pathway plays a key role in many biological and cellular processes, such as cell invasion, migration, and angiogenesis [22, 36, 37]. Several key molecules of PI3K signaling pathway were detected. Under the action of upstream factors, P13K is activated and acts on its substrate PIP2, resulting in its phosphorylation to PIP3, thereby activating mTOR to play its role. The activated mTOR can then lead to the phosphorylation of its downstream translation-inhibiting molecule 4E-BP1, thus promoting protein translation and other synthesis processes, leading to affecting the proliferation, differentiation, and migration of cells [23]. Our study found that ADAMTS12 overexpression in cervical cancer cells upregulated the phosphorylation levels of mTOR and 4E-BP1. Therefore, we speculated that ADAMTS12 activates the PI3K signaling pathway by interacting with proteins in the TGF-β signaling pathway, and then plays a carcinogenic role in cervical cancer. However, there are still many deficiencies in this study. For example, the results of mass spectrometry were not verified by the WB experiment, and whether the glycosylation of ADAMTS12 protein affects the biological function of tumor will be our possible direction of follow-up research.

ADAMTS12-related protein is highly associated with the matrix, which also suggests that the function of ADAMTS12 in tumors may require the cooperation of other matrix components, such as fibroblasts, immune cells, etc. In addition, ADAMTS12 is also a key mediator in the process of inflammation [38], so we chose to analyze the relativity between ADAMTS12 and immune cells. We select non-specific immune cells, CD20 + B cells, CD57 + NK cells, CD68 + M cells, CD163 + M2 cells. The correlation between 4 immune cells and ADAMTS12 was analyzed by Pearson. ADAMTS12 was positively correlated with CD20 + B cells, CD68 + M cells, CD163 + M2 cells. These results also suggest that ADAMTS12 may promote cancer progression by affecting the phosphorylation levels of mTOR and 4E-BP1 in the PI3K signaling pathway, and then induce the infiltration of B cells, macrophages, and other immune cells. TGF-β signaling pathway has been known to affect immune system to promote cervical cancer development [39, 40]. Therefore, ADAMTS12 could affect the PI3K/Akt/mTOR signaling pathway and immune cells via interacting with TGF-β signaling pathway.

In conclusion, this study shows that ADAMTS12 gene is closely related to migration and invasion ability of cervical cancer, and can be used as an indicator of poor prognosis of patients. Further, upregulation of ADAMTS12 gene can affect mTOR signaling pathway, suggesting that the ADAMTS12 gene can provide a new target for diagnosis and treatment of cervical cancer. It also provides a fresh idea and direction for the study of tumor metastasis and invasion.

### Supplementary Information

Supplementary material 1

## Data Availability

The data that support the findings of this study are available from the corresponding author upon reasonable request.
