# Supplementary figures and images for: Effects of metalloprotease ADAMTS12 on cervical cancer cell phenotype and its potential mechanism
Source: Discov Oncol. 2023 Aug 29;14:162. doi: 10.1007/s12672-023-00776-2 (PMC10465472; doi:10.1007/s12672-023-00776-2)

Fig 1G

ADAMTS12

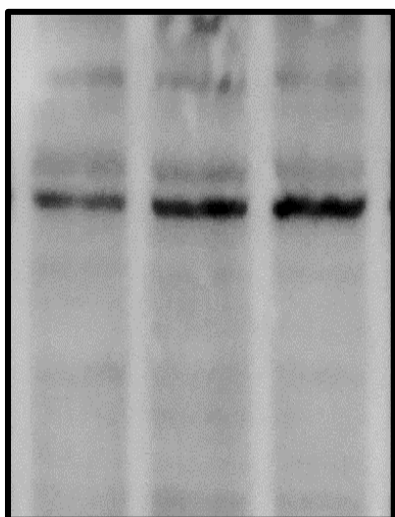

GAPDH

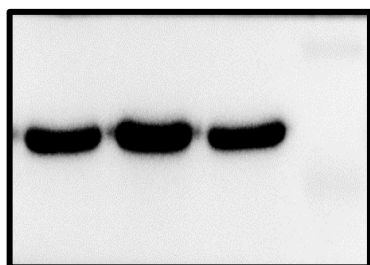

Fig 2D

ADAMTS12

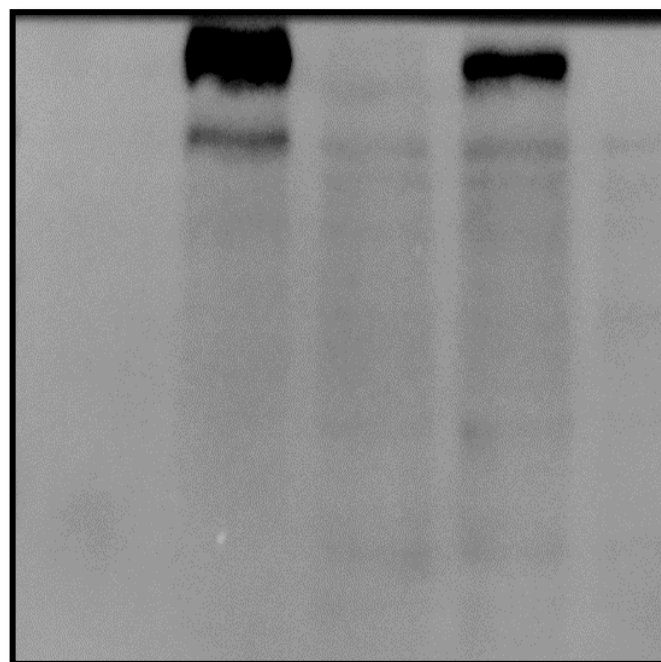

HA

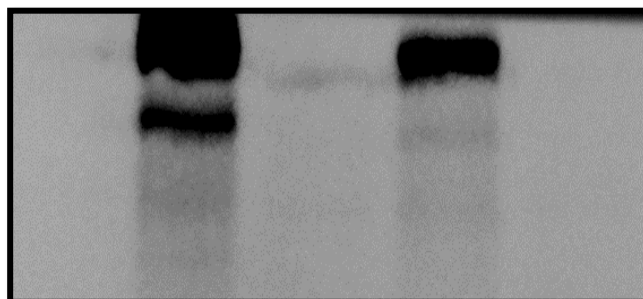

GAPDH

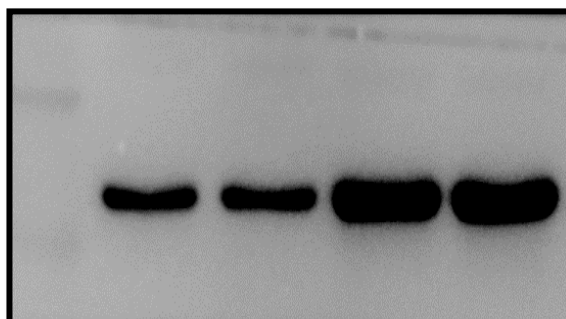

**Fig 2E**

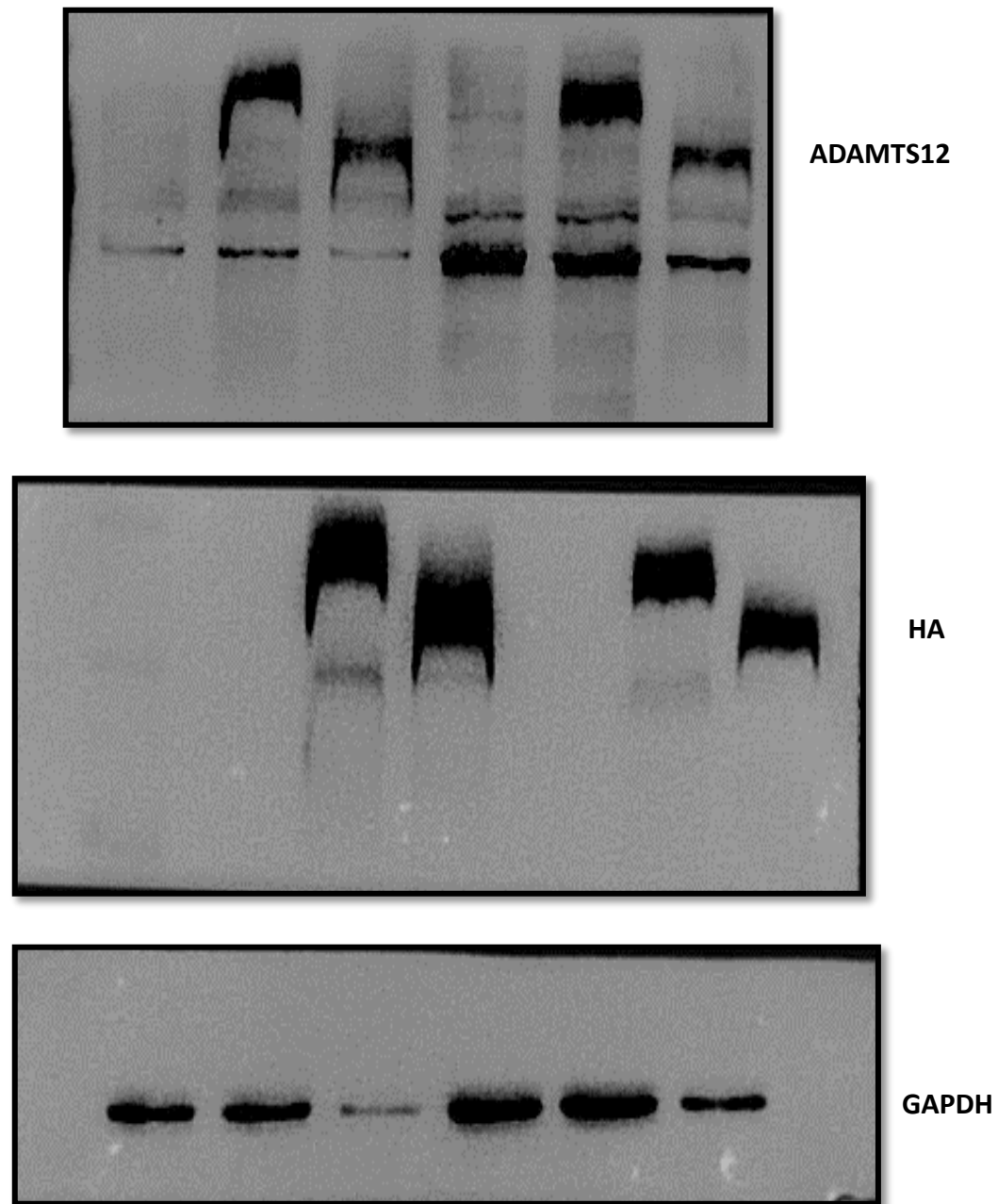

**Fig 5C**

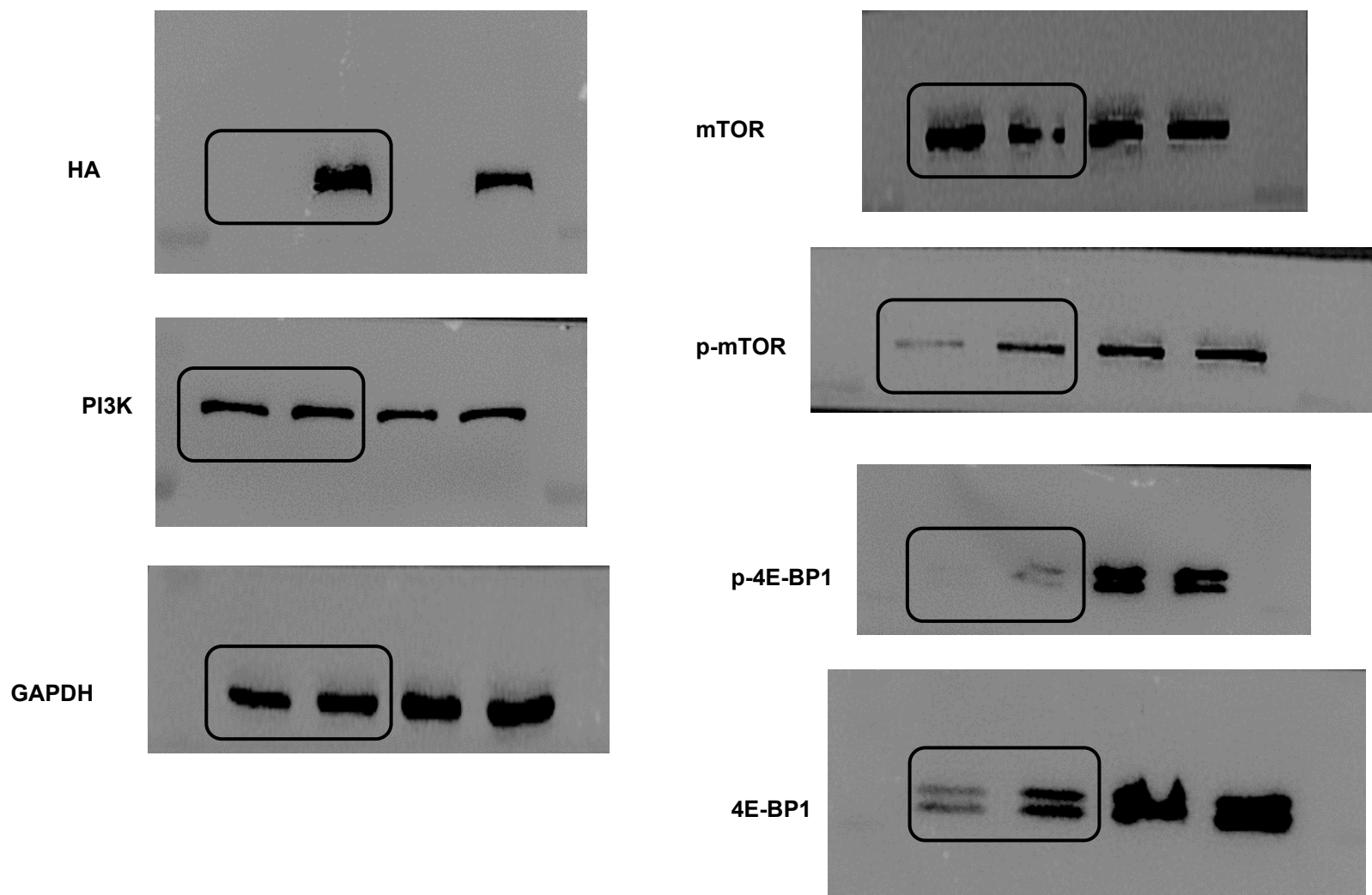

Supplement: Supplementary file 1 — Supplementary material 1 [file 12672_2023_776_MOESM1_ESM.pdf]
